# T1w dark blood imaging improves detection of contrast enhancing lesions in multiple sclerosis

**DOI:** 10.1371/journal.pone.0183099

**Published:** 2017-08-10

**Authors:** Christian Thaler, Tanja Schneider, Jan Sedlacik, Daniel Kutzner, Jan-Patrick Stellmann, Christoph Heesen, Jens Fiehler, Susanne Siemonsen

**Affiliations:** 1 Department of Diagnostic and Interventional Neuroradiology, University Medical Centre Hamburg-Eppendorf, Hamburg, Germany; 2 Department of Neurology, University Medical Centre Hamburg-Eppendorf, Hamburg, Germany; 3 Institute for Neuroimmunology and Clinical MS Research, University Medical Centre Hamburg-Eppendorf, Hamburg, Germany; Universitatsklinikum Freiburg, GERMANY

## Abstract

**Purpose:**

In multiple sclerosis (MS) the sensitivity for detection of contrast enhancing lesions (CEL) in T1-weighted scans is essential for diagnostics and therapy decisions. The purpose of our study was to evaluate the sensitivity of T1w MPRAGE scans in comparison to T1w dark blood technique (T1-DB) for CEL in MS.

**Materials and methods:**

3T MR imaging was performed in 37 MS patients, including T2-weighted imaging, T1w MPRAGE before and after gadolinium injection (unenhanced-T1 and T1-CE) and T1-DB imaging. After gadolinium application, the T1-DB scan was performed prior to T1-CE. From unenhanced-T1 and T1-CE scans, subtraction images (T1-SUB) were calculated. The number of CEL was determined separately on T1-CE and T1-DB by two raters independently. Lesions only detected on T1-DB scans then were verified on T1-SUB. Only lesions detected by both raters were included in further analysis.

**Results:**

In 16 patients, at least one CEL was detected by both rater, either on T1-CE or T1-DB. All lesions that were detected on T1-CE were also detected on T1-DB images. The total number of contrast enhancing lesions detected on T1-DB images (n = 54) by both raters was significantly higher than the corresponding number of lesions identified on T1-CE (n = 27) (p = 0.01); all of these lesions could be verified on SUB images. In 21 patients, no CEL was detected in any of the sequences.

**Conclusions:**

The application of T1-DB technique increases the sensitivity for CEL in MS, especially for those lesions that show only subtle increase in intensity after Gadolinium application but remain hypo- or iso-intense to surrounding tissue.

## Introduction

Magnetic resonance imaging (MRI) is an essential tool in diagnosing and evaluating disease progression in patients with multiple sclerosis (MS). Besides T2 and T1 lesion load, the appearance of contrast enhancing lesions (CEL) is commonly used as a marker for active inflammation and blood brain barrier breakdown and may even predict longterm outcome in patients suffering from MS.[1,2] Furthermore, the detection of CEL has gained importance with revisions of the diagnostic criteria and can be important for treatment decisions.[3,4] The evidence of a CEL can be essential to fulfil the diagnostic criteria for dissemination in time and in later stages the number of CEL has been used as surrogate for insufficient therapeutic suppression of inflammation.

The application of contrast agents is routinely used in diagnosing and monitoring MS as well as in phase I, II and III clinical trials.[5] In clinical routine, two-dimensional T1-weighted spin-echo (T1w-SE) sequences or three-dimensional gradient-echo (T1w-GRE) sequences are commonly acquired for CEL detection in MS patients. However, standardized MR protocols for lesion detection are missing as T1w-SE and T1w-GRE both have their individual limitations.[6–9]

The aim of this study was to introduce T1w dark blood (T1-DB) sequences in CEL detection and to compare this novel method with the routinely used techniques. T1-DB sequences are primarily used for vessel wall imaging and have shown high sensitivity for the detection of vessel wall inflammation, cervical artery dissection and venous thrombosis.[10–13] Furthermore, in recent studies it was demonstrated that at 1.5 Tesla, T1-DB sequences were superior to T1w-SE detecting brain lesions such as primary central nervous system malignant neoplasia and metastases.[14,15] However, these former studies included a small heterogeneous group of potential contrast enhancing lesions and, so far, no study exists examining the potential of T1-DB sequences for CEL detection in a larger homogeneous population or MS. We therefore chose a population of MS patients, where the detection, number and volume of contrast-enhancing lesions is relevant in the diagnostic work-up and is used as endpoint in therapy studies. With the promising results of the recently published studies we hypothesized, that post-contrast T1-DB sequences detect more enhancing MS lesions than the routinely used T1w-GRE with superior inter-observer agreement.

## Materials and methods

### Patients

Thirty-seven patients diagnosed with relapsing-remitting MS were consecutively included in this prospective study between February 2014 and August 2016. Inclusion criteria were as follows: age 18–70 years; diagnosis of relapsing-remitting MS according to the 2010 revised McDonald cirteria [3]; absence of neurologic conditions other than MS. Patients with progressive forms of MS were excluded. All patients were referred to our department from the MS day hospital and received brain MRI. The study was approved by the local Ethical Committee Hamburg (Ethik-Kommission der Ärztekammer Hamburg) following the guidelines of the Declaration of Helsinki and patients provided written informed consent.

### Image acquisition

MR scans were performed on a 3 Tesla MR scanner (Skyra, Siemens Medical Systems, Erlangen, Germany) and the MR protocol included a sagittal 3-dimensional FLAIR (echo-time (TE) = 390 ms, repetition time (TR) = 4700 ms, inversion time (TI) = 1800 ms, acquisition time (TA) = 5:21 min, 192 slices, field of view (FOV) = 256 mm, voxel size = 1.0 x 1.0 x 1.0 mm), a T1-weighted MPRAGE (T1w-MPRAGE) before and after Gadolinium injection (unenhanced T1w and T1-CE) (TE = 2.43 ms, TR = 1900 ms, TI = 900 ms, TA = 4:26 min, 192 slices, FOV = 256 mm, voxel size = 1.0 x 1.0 x 1.0 mm, flip-angle = 9°) and a T1-weighted dark blood (T1-DB) sequence (TE = 3.8 ms, TR = 700 ms, TA = 3:09 min, 192 slices, FOV = 240 mm, voxel size = 0.9 x 0.9 x 0.9 mm, variable flip-angles optimized for T1 contrast). T1w-MPRAGE and T1-DB were both acquired in sagittal plane. All patients received the same Gadolinium based contrast agent, which was injected with a consistent dose of 0.2 ml/kg of body weight. Subsequently, the T1-DB images were acquired 4 minutes after intravenous contrast agent administration, followed by the acquisition of T1-CE (starting approximately 7 minutes after Gadolinium injection).

After image acquisition, unenhanced T1w images were linearly registered to T1-CE images and subtracted afterwards using Analyze 11.0 (AnalyzeDirect, Inc. KS, USA) to obtain a subtraction image (T1-SUB).

### Image analysis

Lesion detection was performed by two independent raters with a specific training of ten years and two years in MS image diagnostics and evaluation. In a first reading, T1-DB and T1-CE images were separately presented to the two raters in random order and contrast enhancing lesions were marked on both images independently. In a second reading, lesions that were identified on T1-DB but not on T1-CE were retrospectively evaluated on T1-CE and on T1-SUB to scan for false positive findings in T1-DB. Hence, all lesions that were detected on T1-DB and not detected on T1-CE were compared to T1-SUB images to confirm contrast enhancement.

For further analysis, only lesions that were identified by both raters in the first reading were included. Lesions were outlined using the software Analyze 11.0 and lesion volumes were calculated as mean of both raters. Subsequently, lesion count and volume were then compared between T1-CE and T1-DB scans using Wilcoxon signed-rank test. Statistical analysis was performed by using Statistics in R 3.0.0 and IBM SPSS 21.0 (IBM Corp., Armonk, NY).

## Results

**Fig 1:**
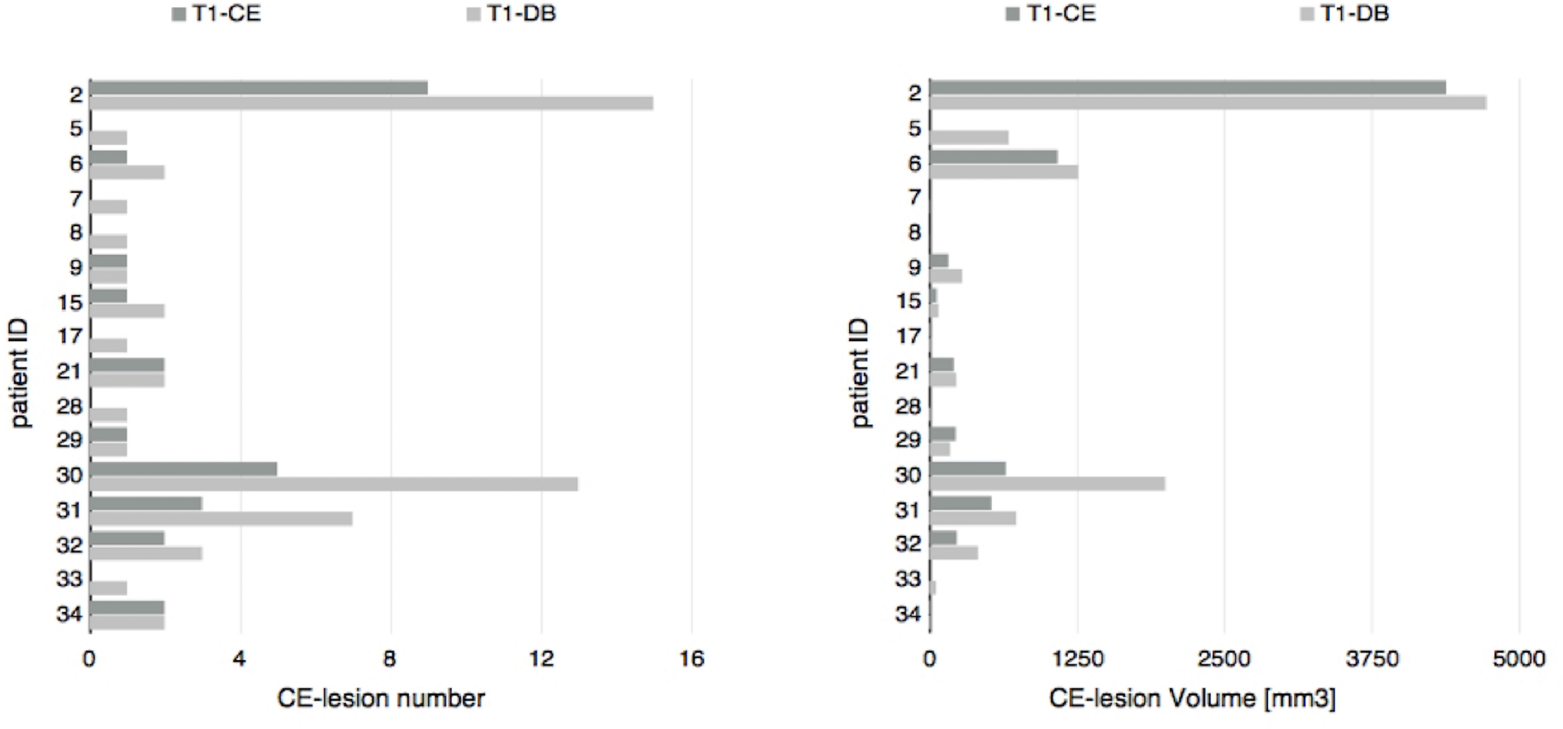
Consensus number and volume of contrast enhancing lesions for each patient in un-subtracted T1w-MPRAGE and T1w dark blood. T1-CE = T1w-MPRAGE after Gadolinium injection, T1-DB = T1w dark blood after Gadolinium injection, CE-lesion = contrast enhancing lesion.

**Fig 2:**
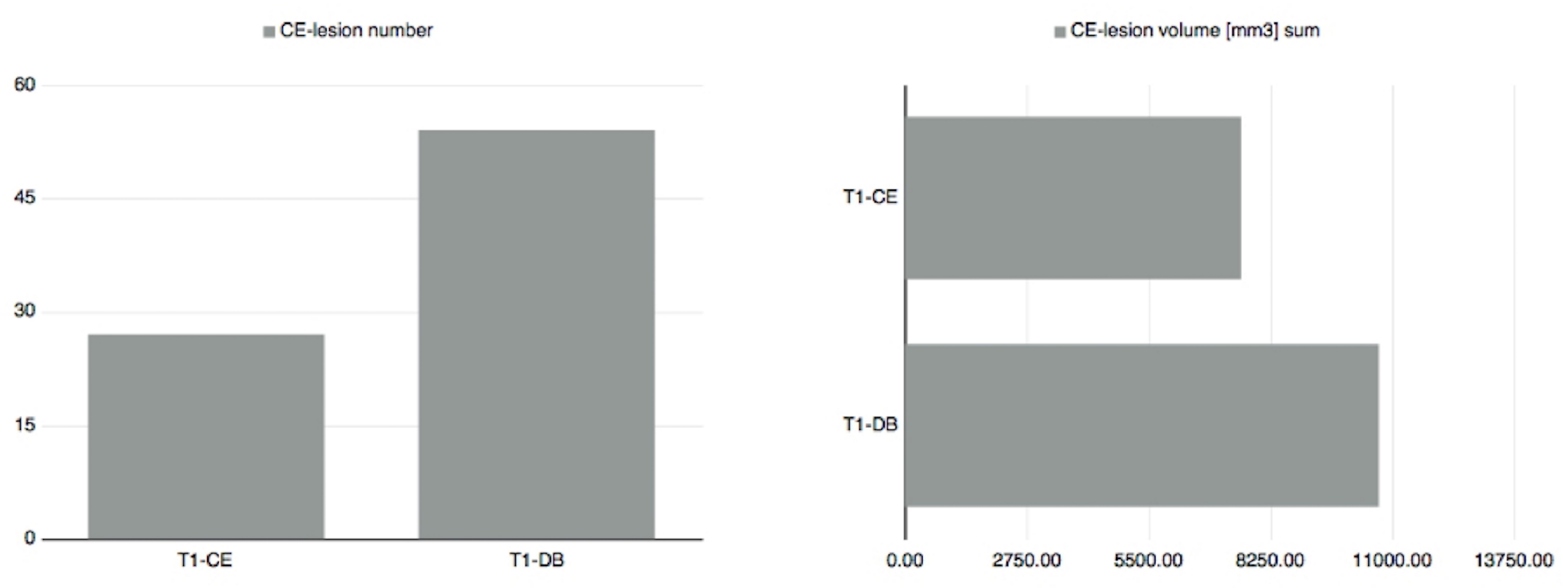
Overall consensus number and volume of contrast enhancing lesions in un-subtracted T1w-MPRAGE and T1w dark blood. T1-CE = T1w-MPRAGE after Gadolinium injection, T1-DB = T1w dark blood after Gadolinium injection, CE-lesion = contrast enhancing lesion.

37 patients (11 male and 26 female) with a mean age of 37.3 years (± 11.7 years) were included in the study. 16 of these patients presented with at least one contrast enhancing lesion detected by both raters, either on T1-CE or T1-DB images. The remaining 21 patients did not show any CEL–neither on T1-CE, nor on T1-DB images. For a detailed overview see Figs 1 and 2.

All lesions that were detected on T1-CE were also detected on T1-DB images. Also, all lesions that were only detected on T1-DB images could retrospectively be confirmed on T1-SUB images.

Raters agreed on the detected number of CE-lesions in 89% for T1-CE and 86% for T1-DB images. All ratings did not differ by more than one count for both sequences.

When only including patients with any CEL on T1-CE or T1-DB images, 54 CE-lesions were detected on un-subtracted T1-DB images and 27 CE-lesions on un-subtracted T1-CE images after consensus reading. Using the Wilcoxon signed-rank test, the number of CEL detected on T1-DB images (median = 1.5) was significantly higher than the corresponding number of lesions identified on T1-CE images (median = 1) (z = 3.21; p = 0.01).

The total CEL volume on un-subtracted T1-DB was 10708 mm^3^ and 7593.5 mm^3^ on un-subtracted T1-CE images. Using the Wilcoxon signed-rank test, CEL volume was significantly higher on T1-DB images (median = 205.5 mm^3^) compared to lesion volume on T1-CE images (median = 114.8 mm^3^) (z = 2.9; p = 0.023)(Figs 1 and 2).

## Discussion

**Fig 3:**
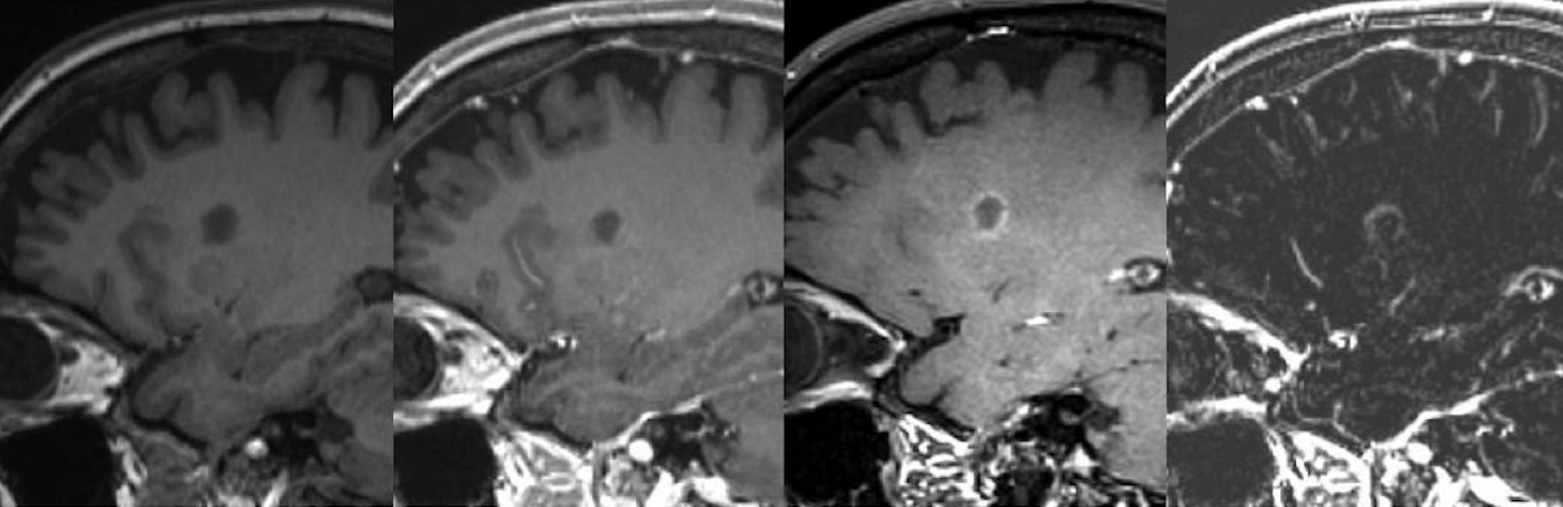
Improved lesion visibility in T1w dark blood image. From left to right: T1w-MPRAGE before Gadolinium injection, T1w-MPRAGE after Gadolinium injection, T1w dark blood after Gadolinium injection, substraction image.

The aim of this study was to introduce post-contrast T1-DB sequences in CEL detection and compare this novel approach with the routinely used T1w-GRE. Our results suggest an increased sensitivity for detecting CEL by using T1-DB sequences. Especially lesions that only show subtle increase in intensity after the application of contrast medium were more reliably detectable in T1-DB than in T1-CE (Fig 3). Therefore, it was possible to identify patients with active inflammatory processes and blood brain barrier breakdown, which would have been missed if lesion detection had only been performed on the routinely acquired sequences. Furthermore, detection of CEL is important as it can be the essential factor to fulfil the diagnostic criteria for dissemination in time and can be important for treatment decisions.

**Fig 4:**
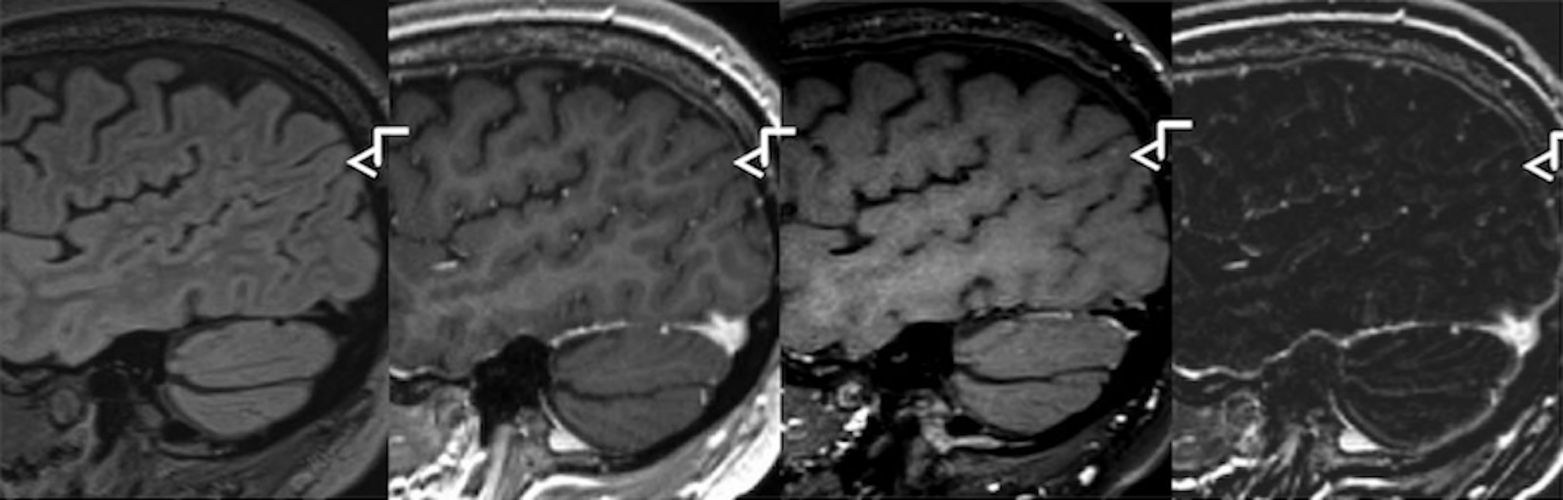
Improved visibility of a juxtacortical lesion in T1w dark blood image. From left to right: FLAIR, T1w-MPRAGE after Gadolinium injection, T1w dark blood after Gadolinium injection, subtraction image.

**Fig 5:**
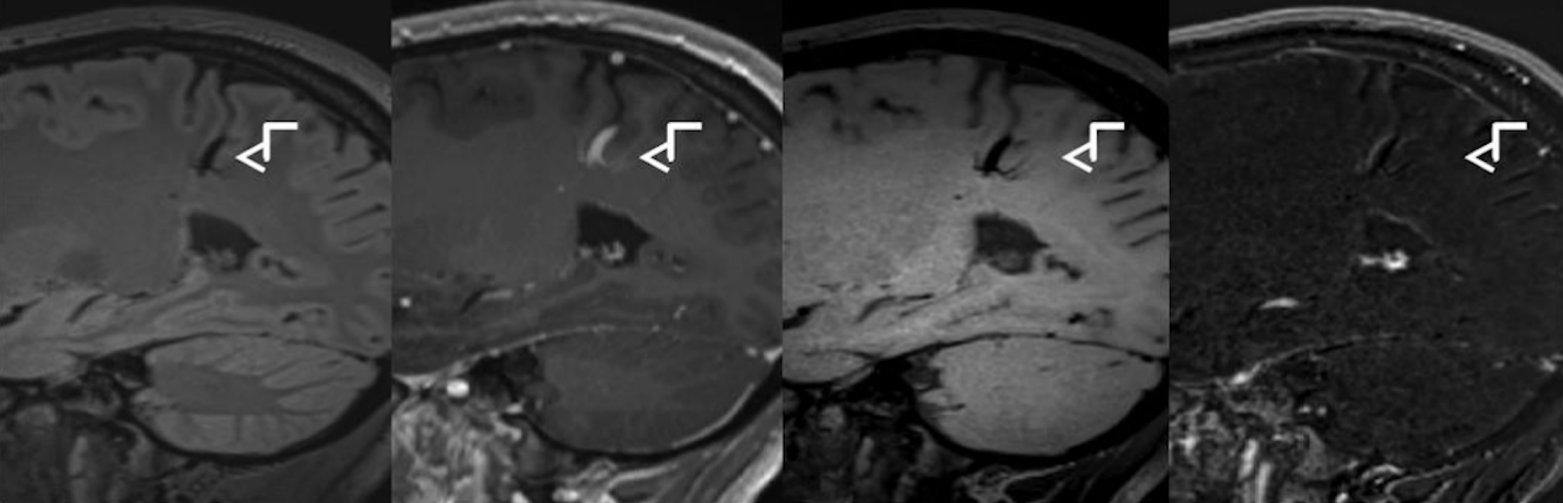
Suppression of vessel in T1w dark blood image. From left to right: FLAIR, T1w-MPRAGE after Gadolinium injection, T1w dark blood after Gadolinium injection, subtraction image.

Due to high-spatial resolution (voxel size = 0.9 mm^3^) and improved lesion detection in T1-DB sequences, a more accurate lesion localization can be achieved (Fig 4). For example, while in FLAIR and T1-CE images a lesion seemed to be located in the periventricular white matter, an involvement of subcortical u-fibres was detected in T1-DB and therefore helped in defining the lesion's location as juxtacortical. In consideration of the 2010 revised McDonald criteria as well as the 2016 introduced Magnetic Resonance Imaging in Multiple Sclerosis (MAGNIMS)-recommended criteria lesion localization is highly important in diagnosing MS, which might induce early therapy decision.[3,4] Furthermore, T1-DB sequences help to minimize false positive findings like arterial or venous vessels by suppression of the blood signal and might therefore prevent unnecessary therapy changes (Fig 5).

Our findings are in line with recent studies, that obtained higher detection rates for brain lesions using T1-DB sequences compared to the routinely used T1w-SE or T1w-GRE sequences.[14,15] However, we did not compare between T1-DB and T1w-SE but T1w-MPRAGE, which is a 3D gradient echo sequence with an initial 180 degree inversion pulse and routinely used in our institution as standard sequence for CEL detection in MS patients. While it is reported that T1w-SE shows better sensitivity of contrast enhancement than T1w-GRE at 1.5 T, it is also prone to flow related artefacts and is usually acquired in thicker slices, since covering the whole brain in thin slices would take too much time in clinical routine.[7,16] Furthermore, it is uncertain whether T1w-SE imaging shows superior contrast intensity than T1w-GRE at higher field strengths.[17] Recent studies reported higher detection rates and reproducibility of contrast enhancing lesions in patients with cerebral tumors and MS, especially those with smaller size, using T1w-GRE at 3 T.[8,9,18] Additionally, T1w-GRE imaging provides whole-brain coverage with thin-section thickness in clinical acceptable scanning times. We used 3D T1w-MPRAGE for lesion detection, which has shown superior detections rates for small enancing brain lesions compared to 2D FLASH sequences.[19] In consideration of these recent findings T1w-MPRAGE seems to be a suitable sequence to compare to.

Recent studies have shown, that serial application of gadolinium-based contrast agents leads to increased signal intensities in certain brain structures, indicating a deposition of gadolinium in the patient's brain.[20–22] Since it remains unclear whether this deposition leads to histopathological damages or health related long-term effects, the application of gadolinium-based contrast agents should be limited to a minimum as possible. With superior detection rates of CEL in T1-DB due to its higher signal-intensity a reduction of contrast agent dose might be possible. However, further investigations and applications of varying doses of contrast agent would be needed.

**Fig 6:**
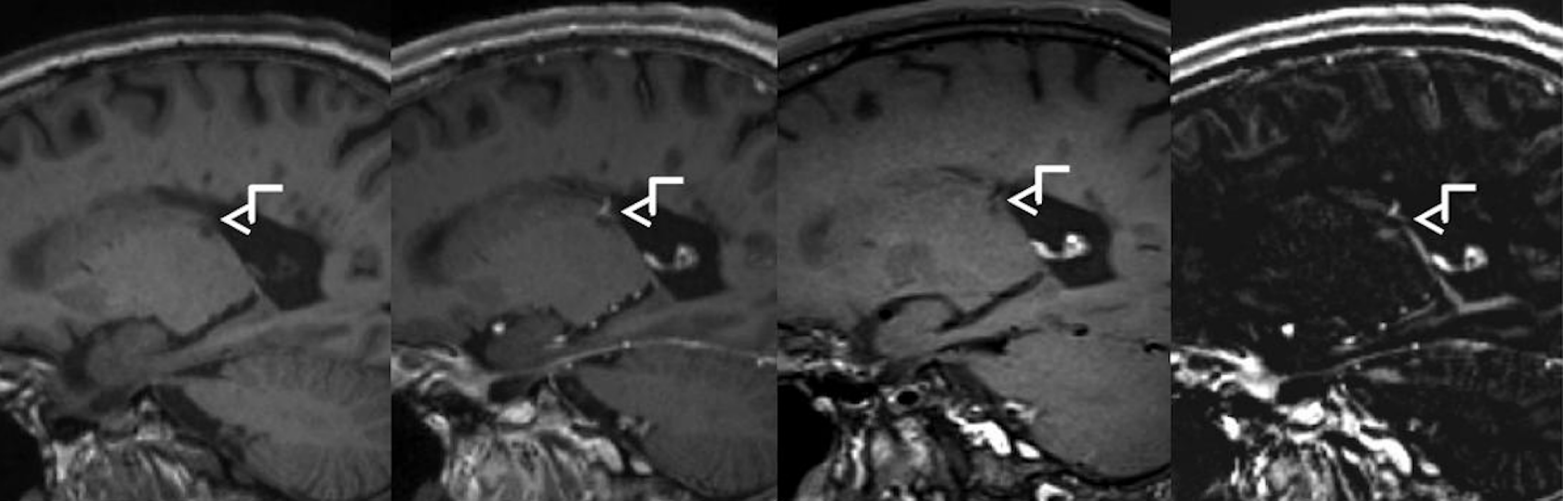
False-positive contrast enhancing lesion in subtraction image. From left to right: T1w-MPRAGE before Gadolinium injection, T1w-MPRAGE after Gadolinium injection, T1w dark blood after Gadolinium injection, subtraction image.

Since detection of CEL is always highly rater dependent, it has been suggested to use subtraction imaging to increase sensitivity in lesion detection, especially for lesion with only subtle contrast enhancement.[23–26] However, automatically processed image registration is not provided by all vendors or workstations on-the-fly. Even if available it still has its limitations and subtraction without prior registration is prone to motion artefacts and might lead to a number of false positive results (Fig 6). Therefore, using only T1-SUB for lesions detection is highly ineffective and is not recommended by any of the recently published consensus guidelines.[3,4,27] In this presented study T1-SUB was used to confirm lesion enhancement in T1-DB and eliminate possible false-positive findings, of which we could not find any. Using T1-DB sequences we achieved superior signal intensity contrast between contrast-enhancement and brain parenchyma but are not dependent on post-processing work steps to avoid false-positive findings due to motion artefacts.

The main limitation in this presented study is the small sample size. Though 37 MS patients received MRI, CEL were detected in only 16 of them, which were subsequently inlcuded in statistical analysis. However, we still obtained significant different detection rates between the two acquisition techniques. Since contrast enhancement is an irregular phenomenon in MS and can be completely absent in MRI, a larger sample size does not automatically assure a higher CEL count. To confirm our results, further studies with larger study cohorts are needed.

Another limitation might be the time between the application of contrast agent and the image acquisition. In our imaging protocol, T1-DB sequences were acquired approximately 4 minutes after the administration of contrast medium but always before acquisition of T1-CE, which was started approximately 7 minutes after Gadolinium injection. Optimal enhancement of lesions is reported 5 to 10 minutes after intravenous injection of the contrast agent.[28] Time for contrast enhancement was always shorter in T1-DB than in T1-CE, but still higher detection rates were obtained for T1-DB. Nevertheless, it would be highly interesting to vary the delay between Gadolinium injection and image acquisition.

In conclusion, T1-DB is a promising MR sequence to increase sensitivity in CEL detection in MS patients, to minimize false-positive findings and finally might enable the reduction of contrast agent dose. Due to its short acquisition time and since no post-processing steps are needed it can be easily established in clinical routine. However, further investigations with larger sample size are needed to confirm our very promising findings.

## Supporting information

S1 File Lesion number and volume for each patient after consensus reading. CE-lesion = contrast enhancing lesion, T1-CE = T1w-MPRAGE after Gadolinium injection, T1-DB = T1w dark blood after Gadolinium injection.
(XLS)

## Abbreviations

CEL: contrast enhancing lesion
unenhanced-T1: T1-weighted MPRAGE before Gadolinium injection
T1-CE: T1-weighted MPRAGE after Gadolinium injection
T1-SUB: T1-weighted substraction image
T1w-GRE: T1-weighted gradient-echo
T1w-SE: T1-weighted spin-echo
